# PTEN Mediates the Antioxidant Effect of Resveratrol at Nutritionally Relevant Concentrations

**DOI:** 10.1155/2014/580852

**Published:** 2014-04-10

**Authors:** Marta Inglés, Juan Gambini, M. Graça Miguel, Vicent Bonet-Costa, Kheira M. Abdelaziz, Marya El Alami, Jose Viña, Consuelo Borrás

**Affiliations:** ^1^Department of Physiotherapy, Faculty of Physiotherapy, University of Valencia, Gascó Oliag Street 5, 46010 Valencia, Spain; ^2^Department of Physiology, Faculty of Medicine, University of Valencia, Blasco Ibañez Avenue 15, 46010 Valencia, Spain; ^3^Centro Biotecnologia Vegetal, Faculdade de Ciências e Tecnologia, Instituto de Biotecnologia e Bioengenharia, Universida de do Algarve, Campus de Gambelas, Edifício 8, 8005-139 Faro, Portugal

## Abstract

*Introduction.* Antioxidant properties of resveratrol have been intensively studied for the last years, both *in vivo* and *in vitro*. Its bioavailability after an oral dose is very low and therefore it is very important to make sure that plasma concentrations of free resveratrol are sufficient enough to be active as antioxidant. *Aims.* In the present study, using nutritionally relevant concentrations of resveratrol, we aim to confirm its antioxidant capacity on reducing peroxide levels and look for the molecular pathway involved in this antioxidant effect. *Methods.* We used mammary gland tumor cells (MCF-7), which were pretreated with different concentrations of resveratrol for 48 h, and/or a PTEN inhibitor (bpV: bipy). Hydrogen peroxide levels were determined by fluorimetry, PTEN levels and Akt phosphorylation by Western Blotting, and mRNA expression of antioxidant genes by real-time reverse transcriptase-polymerase chain reaction (RT-PCR). *Results.* Resveratrol treatment for 48 h lowered peroxide levels in MCF-7, even at low nutritional concentrations (1 nM). This effect was mediated by the activation of PTEN/Akt pathway, which resulted in an upregulation of catalase and MnSOD mRNA levels. *Conclusion.* Resveratrol acts as an antioxidant at nutritionally relevant concentrations by inducing the expression of antioxidant enzymes, through a mechanism involving PTEN/Akt signaling pathway.

## 1. Introduction

In the last two decades, life expectancy at birth has increased by 5–10 years [1]. As a consequence, the population is growing older and therefore there is increasing interest in how to face age-related problems. Oxidative damage to biomolecules caused by reactive oxygen species (ROS) plays an important role in the aging process, as stated in the free radical theory of aging [2]. This occurs when an imbalance between the production of free radicals and the ability of the natural antioxidant defenses to scavenge them exists. We have previously suggested the possibility that the intake of nonvitamin antioxidants such as nutrients or natural compounds may be effective in increasing antioxidant defenses, by upregulating the activity of antioxidant enzymes, which are normally present in cells [3]. One of these plausible antioxidants is resveratrol (*trans*-3,5,4′-trihydroxystilbene), a natural polyphenol found in many plants and fruits, such as blueberries, blackberries, peanuts, and grapes, and specially in red wine, the main source in the human diet [4]. Resveratrol properties as an antioxidant have been intensively studied for the last years [5–10], both* in vivo* and* in vitro*, but the mechanism involved remains unclear. Moreover, its bioavailability after an oral dose is very low. In fact, plasma concentrations of free* trans*-resveratrol after ingestion of 600 mL of red wine [11] or an oral dose of 25 mg [12, 13] are extremely low, of the order of nanomolar or low micromolar. Therefore, it is very important to make sure that those nutritionally relevant low plasma concentrations of free resveratrol are sufficient enough to be active as an antioxidant [13, 14].

One of the antioxidant effects attributed to resveratrol is the ability to protect cells against H_2_O_2_-induced oxidative stress [15, 16]. Furthermore, resveratrol is able to increase the phosphatase and tensin homolog PTEN [17, 18], a well-known tumor suppressor that antagonizes the activity of phosphatidylinositol 3-kinase type I (PI3K), thus leading to decreased phosphorylated-Akt (P-Akt) levels [19]. Interestingly, it has been recently reported that PTEN is able to increase energy expenditure and improve organismal survival independently of its effect on cancer, thus suggesting that PTEN might have multiple protective functions [20].

We have previously reported that oestradiol and genistein are able to decrease hydrogen peroxide levels in MCF-7 cells [21, 22]. Using the same cell line, we report that nutritionally relevant concentrations of resveratrol are able to decrease hydrogen peroxide levels not because of its phenolic structure, but because of the fact that it induces the expression of antioxidant genes, such as catalase (Cat) and manganese superoxide dismutase (MnSOD), through a mechanism that involves phosphatase and tensin homolog (PTEN) and protein kinase-B (PKB or Akt) signaling pathway. This finding may be useful to support the idea that, despite having low bioavailability, it is possible to consider resveratrol as an important nonvitamin antioxidant and to provide new insights into the mechanism involved in it.

## 2. Results

### 2.1. Nutritional Concentrations of Resveratrol Decrease Hydrogen Peroxide Levels in MCF-7 Cells

**Figure 1:**
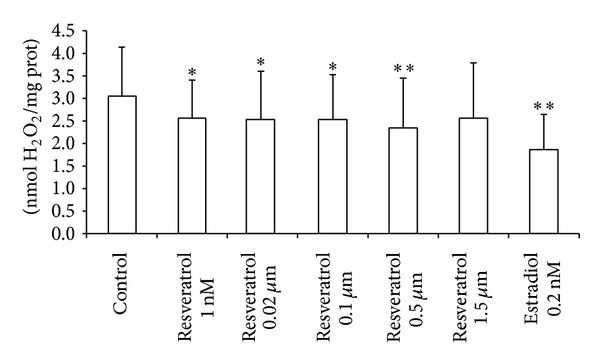
Resveratrol diminishes hydrogen peroxide levels in MCF-7 cells. Peroxide levels were determined by fluorimetry using homovanillic acid (see Section 3). Cells were treated with resveratrol or with estradiol for 48 h. Data are expressed as means + SD for 15 different experiments; *P < 0.05; **P < 0.01* versus* control.

Figure 1 shows that resveratrol treatment for 48 h lowers hydrogen peroxide levels, except at the highest dose (1.5 *μ*M). These concentrations are similar to those of free* trans*-resveratrol found in plasma after ingestion of 600 mL of red wine [11] or an oral dose of 25 mg [12, 13]. Therefore, we find antioxidant effects of resveratrol at nutritionally relevant concentrations.

### 2.2. Resveratrol Increases PTEN Protein Levels in MCF-7 Cells

**Figure 2:**
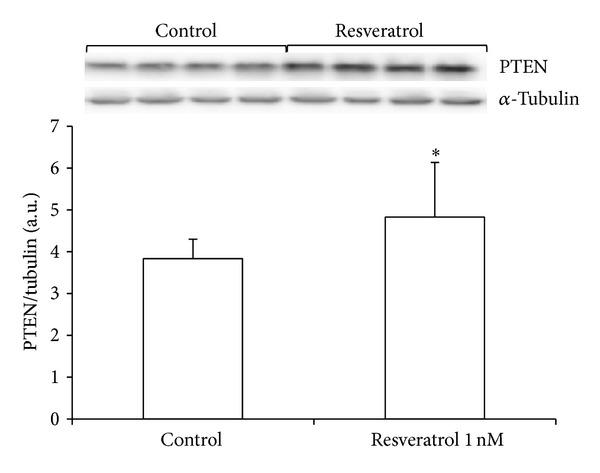
Resveratrol activates the PTEN signaling pathway. Levels of PTEN were measured in cells treated for 48 h with resveratrol (1 nM). Data are expressed as means + SD for 4 different experiments; *P < 0.05* versus* control.

In order to find out the mechanism by which resveratrol acts as an antioxidant, we tested if PTEN signaling pathway could be involved in its antioxidant effect. We found that resveratrol treatment for 48 h increased PTEN protein levels in MCF-7 (Figure 2).

### 2.3. Resveratrol Decreases Akt Phosphorylation via PTEN Activation in MCF-7 Cells

**Figure 3:**
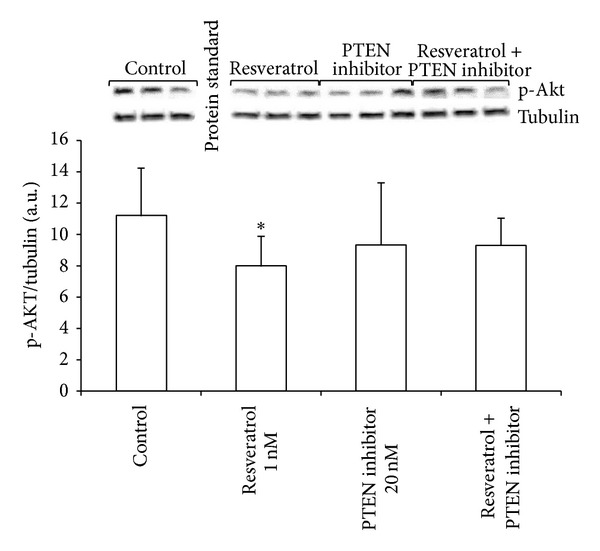
Resveratrol inactivates the Akt signaling pathway through PTEN activation in MCF-7 cells. Phospho-Akt levels were measured by Western blotting, after 48 h incubation with resveratrol (1 nM) alone, potassium bisperoxo (bipyridine) oxovanadate (V) as a PTEN inhibitor alone (20 nM), or both together. Histograms represent densitometric measurement of specific bands of phospho-Akt content using tubulin levels as housekeeping control. Data are expressed as means + SD for 4 independent experiments; *P < 0.05* versus* control.

Figure 3 shows that incubation of MCF-7 cells with physiological concentrations of resveratrol (1 nM) for 48 h reduces the phosphorylation of Akt. This effect can be seen within 5 minutes of incubation and reaches the maximum within 48 h. Coincubation with an inhibitor of PTEN activity (potassium bisperoxo (bipyridine) oxovanadate (V)) at a dose of 20 nM reverts this effect. Therefore, the decrease in Akt phosphorylation by resveratrol is mediated by PTEN activation.

### 2.4. Resveratrol Upregulates Endogenous Antioxidant Genes via PTEN Signaling Pathway

**Figure 4:**
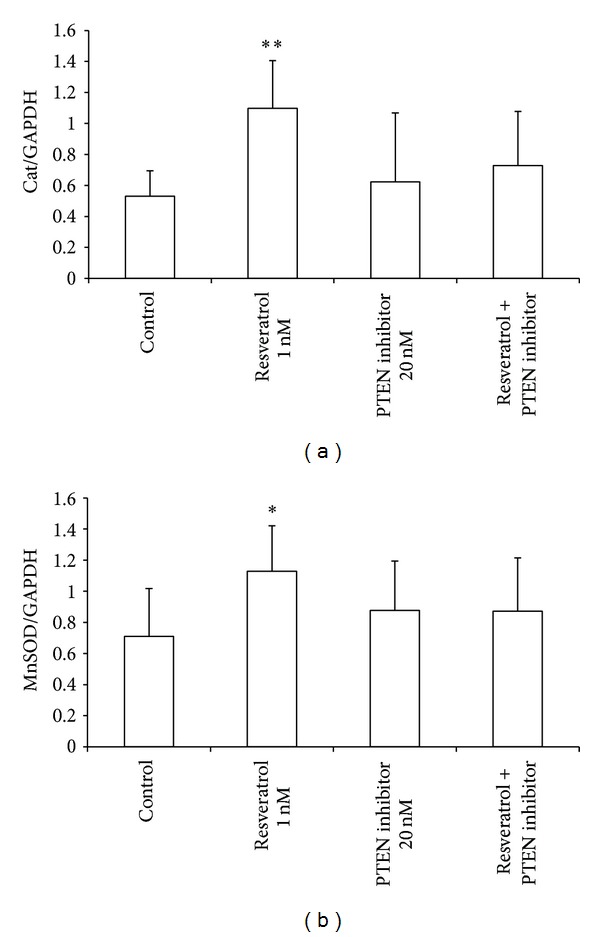
Resveratrol upregulates the expression of catalase (a) and MnSOD (b) in MCF-7 cells. Resveratrol (1 nM) increased mRNA levels of catalase and Mn-superoxide dismutase (*P < 0.05; **P < 0.01* versus* control), and these effects were prevented when coincubating with the PTEN inhibitor (20 nM). Data are expressed as means + SD for 3 different experiments.

Nutritional concentrations of resveratrol upregulate the expression of catalase (Figure 4(a)) and MnSOD (Figure 4(b)) after 48 h of incubation. However, this upregulation is prevented when cells are coincubated with a PTEN inhibitor, suggesting the implication of PTEN in resveratrol-mediated activation of endogenous antioxidant gene expression.

### 2.5. PTEN Mediates the Antioxidant Effect of Resveratrol in MCF-7 Cells

**Figure 5:**
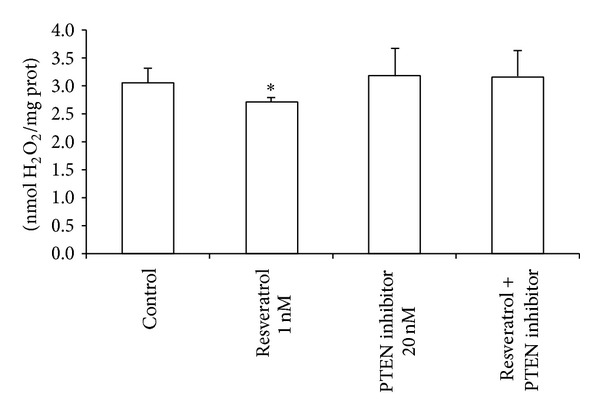
Resveratrol diminishes peroxide levels in MCF-7 cells, and this effect is mediated by the PTEN/Akt signaling pathway. MCF-7 cells were treated with resveratrol (1 nM) alone, potassium bisperoxo (bipyridine) oxovanadate (V) as a PTEN inhibitor alone (20 nM), or both together. Data are expressed as means + SD for 4–8 different experiments; *P < 0.05* versus* control.

As stated before, 1 nM resveratrol pretreatment led to a reduction in intracellular hydrogen peroxide levels. However, Figure 5 shows how coincubation with a PTEN inhibitor (20 nM) reverts this antioxidant effect.

## 3. Materials and Methods

### 3.1. Cell Culture

Human mammary gland tumor cells (MCF-7) were cultured in Iscove's modified Dulbecco's medium (IMDM) without phenol red, supplemented with 10% (v/v) heat-inactivated fetal bovine serum. Cells were plated in 25 or 75 cm² culture flasks and maintained at 37°C with 5% CO_2_ in air. All the experiments were performed once cells reached confluence.

### 3.2. Treatments

Based on previous experiments of our laboratory [21, 22] and on the literature [17, 18], cells were treated for 48 h with either DMSO (for the control group), resveratrol (at concentrations ranging from 1 nM to 1.5 *μ*M), or resveratrol together with 20 nM bisperoxo (bipyridine) oxovanadate (V) as PTEN inhibitor [23]. 0.2 nM estradiol was used as a positive control when measuring hydrogen peroxide levels [21].

### 3.3. Determination of Peroxide Levels in MCF-7 Cells

Intracellular levels of hydrogen peroxide were determined by fluorimetry using a modification of the method described by Barja [24].

Briefly, cells were washed twice with PBS and then incubated at 37°C with a PBS solution containing 0.1 mM homovanillic acid and 6 U/mL horseradish peroxidase. The incubation was stopped at 5 min with 1 mL of cold 2 M glycine buffer containing 50 mM EDTA and 2.2 M NaOH. The fluorescence of supernatants was measured using 312 nm as an excitation wavelength and 420 nm as an emission wavelength. The levels of peroxides were calculated using a H_2_O_2_ standard curve and results were expressed per milligram of protein.

### 3.4. Immunoblot Analysis of Akt Phosphorylation and PTEN Protein Expression Levels

After 48 h of pretreatment with resveratrol, cells were washed twice with cold PBS and lysed in cold lysis buffer (62.5 mM Tris-HCl (pH 6.8 at 25°C), 2% w/v SDS, 10% v/v glycerol), which was supplemented with a protease inhibition cocktail (10 *μ*L per 1 mL of lysis buffer) and sodium orthovanadate 200 mM (10 *μ*L per 1 mL of lysis buffer) to inactivate proteases and phosphatases. Immediately after harvesting, aliquots of whole cell lysates (40 *μ*g, based on previous experiments [21, 22]) were boiled for 10 min, electrophoresed on SDS 10% polyacrylamide gels, and electroblotted (Bio-Rad) onto a PVDF membrane (Bio-Rad). Membranes were blocked at room temperature for 1 hour with 0.05 g/mL nonfat milk or BSA 0.05 g/mL in TBS-0.1% Tween 20 (TBS-T) according to the antibody. Afterwards, membranes were incubated with primary antibodies against phospho-Akt Ser 473, PTEN (1 : 1000, Cell Signaling Technologies, Boston, MA, USA), or *α*-tubulin as loading control (1 : 1000, Santa Cruz BioTech USA), overnight at 4°C. Blots were then washed again three times for 10 min at room temperature and then incubated for 1 h with a secondary horseradish peroxidase (HRP) linked anti-rabbit IgG antibody (1 : 2000) (Cell Signaling, Boston, MA, USA). After washing three times again, membranes were developed by using the ECL Prime Western Blotting Detection reagent as specified by the manufacturer (Amersham Pharmacia, USA). Autoradiographic bands were assessed using a Fujifilm scanning densitometer (Fujifilm LAS-1000 plus). The densitometric analysis was performed using Image J 1.34s software. For comparison between blots, one aliquot of the same sample was loaded as a standard in each gel to allow data normalization.

### 3.5. mRNA Gene Expression

Catalase (Cat) and manganese superoxide dismutase (MnSOD) mRNA expression was determined by real-time PCR with glyceraldehyde-3P-dehydrogenase (GAPDH) as the endogenous control, according to previously published results [21, 22].

For this purpose, total RNA was isolated from cultures by extraction with TRIzol Reagent (Invitrogen), according to the manufacturer's instructions. RNA was quantified by measuring the absorbance at 260 nm. The purity of the RNA preparations was assessed by the 260/280 ratio.

cDNA was synthesized from 1 *μ*g total RNA using a reverse transcriptase (RT) system kit of Applied Biosystems (High-Capacity cDNA Reverse Transcription Kits). The reaction was incubated as recommended by the manufacturer, for 10 min at 25°C, followed by 120 min at 37°C, and then for 5 min at 85°C, and finally cooled to 4°C to collect the cDNA and then stored at −20°C prior to the real-time PCR assay.

The quantitative PCR was performed using the detection system 7900HT Fast Real-Time PCR System (Applied Biosystems) with Maxima SYBR Green/ROX qPCR Master Mix (2X) (Fermentas). Target and control were run in separate wells.

Specific primers employed, sense and antisense for each gene, respectively, were MnSOD, 5′-CGT GCT CCC ACA CAT CAA TC-3′ and 5′-TGA ACG TCA CCG AGG AGA AG-3′; Catalase, 5′-ACG TTG GAT GGA GAA GTG CGG AGA TTC AAC-3′ and 5′-ACG TTG GAT GTT CAC ATA GAA TGC CCG CAC-3′; and GAPDH, 5′-CCT GGA GAA ACC TGC CAA GTA TG-3′ and 5′-GGT CCT CAG TGT AGC CCA AGA TG-3′. Target cDNAs were amplified in separated tubes using the following procedure: 10 min at 95°C and then 40 cycles of denaturation at 95°C for 15 s and annealing and extension at 62°C for 1 min per cycle.

The standard curve method was used to evaluate the relative expression levels of catalase and MnSOD in resveratrol pretreated MCF-7 cells. Briefly, the threshold cycle (Ct) was determined and converted to a relative amount through the use of a standard curve prepared from dilutions of cDNA mix of all samples. The logarithmic formula used to transform Ct values was

(1) $$\begin{array}{c} \text{Exp}=\frac{\left(\text{Ct},\text{sample}-\text{Intercept}\right)}{\text{Slope}}. \end{array}$$

### 3.6. Statistical Analysis

Quantitative variables are expressed as means and standard deviation of different experiments. Once the normality of the variables was tested by Kolmogorov-Smirnov test, the statistical analysis was performed using the one-way analysis of variance (ANOVA) test to check any possible statistically significant difference between groups and the adequate post hoc tests. The level of significance was chosen at *P* < 0.01 or *P* < 0.05. All analyses were performed using SPSS statistical software version 19.0.

## 4. Discussion

Antioxidant supplementation is a common medical practice among the elderly [25]. We report here that the antioxidant effect of low nanomolar concentrations of resveratrol is mediated via the upregulation of antioxidant gene expression, involving activation of PTEN/Akt signaling pathway.

Numerous studies have reported the beneficial antioxidant properties of resveratrol. For example, in human blood platelets treated with peroxynitrite, resveratrol inhibited protein carbonylation and nitration, as well as lipid peroxidation [6]. Resveratrol has also been shown to protect primary hepatocytes in culture against oxidative stress damage by increasing the activities of catalase, superoxide dismutase, glutathione peroxidase, NADPH quinine oxidoreductase, and glutathione-S-transferase [7]. In addition, resveratrol was able to diminish oxidative stress by increasing gastrocnemius catalase activity, MnSOD activity, and MnSOD protein content in young and old rats submitted to a 14-day muscle disuse by hindlimb suspension [9].

In our cellular model, resveratrol also acts as an antioxidant by increasing MnSOD and Cat mRNA levels, which in turn decreases H_2_O_2_ levels. This H_2_O_2 _decrease is in agreement with studies reporting the ability of resveratrol to protect PC12 cells against H_2_O_2_-induced cytotoxicity [15, 16], suggesting the potential capacity of resveratrol to prevent oxidative stress-induced cell death. In this regard, we checked the potential antiapoptotic effect of 1 nM resveratrol on MCF-7 cells by measuring BCL-XL levels by Western Blotting. However, we did not find any difference in BCL-XL levels between control and resveratrol group (data not shown), suggesting that the antioxidant effect of such a low dose of resveratrol does not affect apoptosis.

In any case, the mechanism by which resveratrol can exert its antioxidant effect has not been fully elucidated. Resveratrol is able to act as a phytoestrogen and mimic estrogen biological effects [26, 27]. However, it has also been shown to act as an antagonist [28]. In fact, our first experiments aimed to check if resveratrol was able to behave as estradiol in our model system, thus binding to estrogen receptors. However, we could not inhibit resveratrol antioxidant effect when coincubating cells with resveratrol and tamoxifen (an inhibitor of estrogen receptors) (data not shown). Furthermore, resveratrol did not activate MAPK and NF*κ*B signalling pathways, thus suggesting that the pathway involved in the expression of antioxidant genes is not mediated by estrogen receptors or MAPK and NF*κ*B signalling pathways. Interestingly, these results suggest that resveratrol antioxidant effects may not change between men and women.

Here we show that PTEN, an antagonist to the PI3K/Akt pathway, is involved in resveratrol antioxidant effects. Resveratrol increases PTEN and decreases phospho-Akt levels. In this regard, Waite et al. observed that preincubation with resveratrol or other phytoestrogens for 48 h had been able to increase PTEN levels and decrease phospho-AKT levels in MCF-7, at concentrations ranging from 0.1 nM to 1 *μ*M [17]. Wang et al. also found an increase in PTEN levels when incubating a prostate cancer cell line with 10 *μ*M resveratrol for 24 h [18]. Interestingly, PTEN has been recently shown to increase the activity of antioxidant enzymes, such as glutathione peroxidase (GPx), Cat, and MnSOD in a lung cancer cell line [29]. This led us to hypothesize and finally demonstrate that PTEN/Akt pathway was involved in resveratrol antioxidant effect.

As stated before, resveratrol bioavailability is very low and its absorption is highly variable, depending on the way it is consumed and the kind of food ingested [12, 13]. Two of the first human studies on the absorption and bioavailability of resveratrol used a single oral dose treatment of 25 mg [12, 13]. Despite the use of high sensitivity methods and a specific molecular analysis, the presence of nonmetabolized resveratrol in circulating plasma was difficult to detect. Approximate calculations showed maximal concentrations of <10 ng/mL (*≈*40 nM), 0.5–2 hours after the oral dose. Estimates of the plasma concentrations of resveratrol plus total metabolites were considerably higher, around 400–500 ng/mL (*≈*2 *μ*M), indicating a very low oral bioavailability of free resveratrol, but significant one of its metabolites. Vitaglione et al. also studied the bioavailability of resveratrol after red wine consumption and found low micromolar (1–6 *μ*M) or nanomolar concentrations of free* trans-*resveratrol in plasma [11]. Thus, we chose for our experiment the lowest concentration (1 nM) that was able to diminish hydrogen peroxide levels (see Figure 1). This is indeed within the range of nutritionally relevant concentrations found in plasma after moderate wine intake. Consuming normal amounts of resveratrol-rich nutrients, such as grapes, peanuts, blueberries, blackberries, and red wine [30], may result in plasma concentrations of free resveratrol that, as we show here, increase the expression of antioxidant genes and thus may delay the onset of oxidative stress-related conditions. Therefore, our results may have practical importance.

## 5. Conclusions

**Figure 6:**
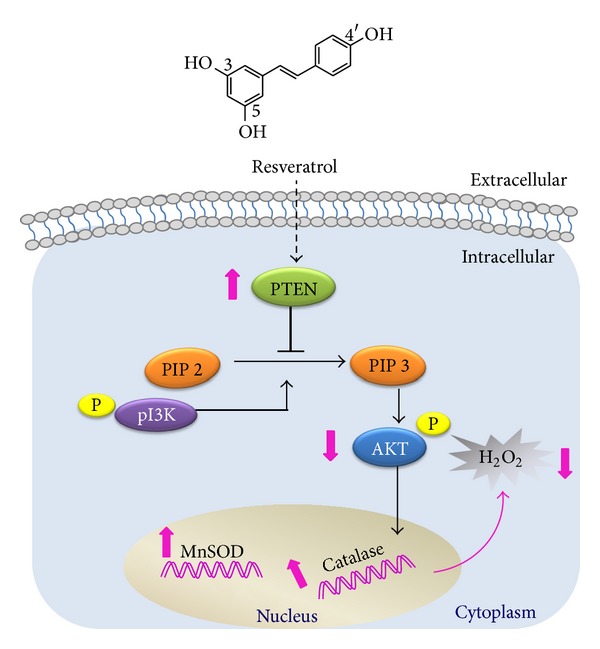
Proposed mechanism for resveratrol to upregulate antioxidant gene expression.

The major conclusion of the current study is that nutritionally relevant concentrations of resveratrol can decrease oxidative stress within the cell by upregulating antioxidant genes. As illustrated in Figure 6, resveratrol increases PTEN levels, which in turn inhibits phosphoinositide 3-kinase (PI3K) function, leading to a decrease in phospho-Akt levels and, finally, to upregulation of antioxidant genes (Cat and MnSOD). As a consequence, lower levels of hydrogen peroxide can be observed within the cell.
